# Systems Approaches to Treatment Response to Imatinib in Severe Asthma: A Pilot Study

**DOI:** 10.3390/jpm11040240

**Published:** 2021-03-25

**Authors:** Seung Han Baek, Dinah Foer, Katherine N. Cahill, Elliot Israel, Enrico Maiorino, Annika Röhl, Joshua A. Boyce, Scott T. Weiss

**Affiliations:** 1 Channing Division of Network Medicine, Brigham and Women’s Hospital and Harvard Medical School, Boston, MA 02115, USA; shbaek@bwh.harvard.edu (S.H.B.); emaiorino@bwh.harvard.edu (E.M.); annika.roehl@gmx.net (A.R.); scott.weiss@channing.harvard.edu (S.T.W.); 2 Division of Allergy and Clinical Immunology, Department of Medicine, Brigham and Women’s Hospital and Harvard Medical School, Boston, MA 02115, USA; eisrael@bwh.harvard.edu (E.I.); jboyce@rics.bwh.harvard.edu (J.A.B.); 3 Division of Allergy, Pulmonary and Critical Care Medicine, Department of Medicine, Vanderbilt University Medical Center, Nashville, TN 37232, USA; katherine.cahill@vumc.org; 4 Division of Pulmonary and Critical Care Medicine, Department of Medicine, Brigham and Women’s Hospital and Harvard Medical School, Boston, MA 02115, USA

**Keywords:** personalized medicine, asthma subtypes, personalized perturbation profiles, systems biology, mitochondria, pharmacogenetics

## Abstract

There is an acute need for advances in pharmacologic therapies and a better understanding of novel drug targets for severe asthma. Imatinib, a tyrosine kinase inhibitor, has been shown to improve forced expiratory volume in 1 s (FEV_1_) in a clinical trial of patients with severe asthma. In a pilot study, we applied systems biology approaches to epithelium gene expression from these clinical trial patients treated with imatinib to better understand lung function response with imatinib treatment. Bronchial brushings from ten imatinib-treated patient samples and 14 placebo-treated patient samples were analyzed. We used personalized perturbation profiles (PEEPs) to characterize gene expression patterns at the individual patient level. We found that strong responders—patients with greater than 20% increase in FEV_1_—uniquely shared multiple downregulated mitochondrial-related pathways. In comparison, weak responders (5–10% FEV_1_ increase), and non-responders to imatinib shared none of these pathways. The use of PEEP highlights its potential for application as a systems biology tool to develop individual-level approaches to predicting disease phenotypes and response to treatment in populations needing innovative therapies. These results support a role for mitochondrial pathways in airflow limitation in severe asthma and as potential therapeutic targets in larger clinical trials.

## 1. Introduction

Severe asthma is a major global cause of morbidity and healthcare costs [1]. More than 60% of adults with asthma, have uncontrolled, symptomatic asthma [2]. There is an acute need for advances in pharmacologic therapies and pharmacogenetics in asthma to improve the identification of novel drug targets [3]. In a randomized clinical trial repurposing imatinib, a tyrosine kinase inhibitor of the KIT proto-oncogene receptor tyrosine kinase, for severe asthma, imatinib use improved forced expiratory volume in 1 s (FEV_1_) compared to placebo treatment [4]. KIT is the receptor for stem cell factor; soluble stem cell factor levels are increased in the serum of asthmatics and correlate with asthma severity [5]. In that trial, imatinib-treated patients with the greatest increases in FEV_1_ had higher baseline bronchoalveolar lavage neutrophil counts, suggesting that imatinib may particularly benefit non-eosinophilic asthmatics.

Patients with non-eosinophilic asthma phenotypes report poor disease control despite multiple controller medications and have limited treatment options [6]. Despite significant advances in asthma treatment over the past decade, biomarkers and available biologics (e.g., anti-IgE, anti-interleukin (IL)-5, anti-IL-4R) are primarily directed to eosinophilic phenotypes [7]. Therefore, clinicians face a dual challenge in treating non-eosinophilic asthma: lack of data-guided therapy and limited therapeutic interventions.

Systems biology analytic approaches are designed to capture complex biological interactions that may inform a particular clinical phenotype [8]. The application of these quantitative tools can identify biological pathways or components of those pathways that predict phenotypes/endotypes, biomarkers and drug interventions [3]. Clinically meaningful findings may contribute to personalized approaches to care. In this pilot study, we conducted a network analysis of bronchial epithelium gene expression to characterize the phenotypes of severe asthma patients with, and without, lung function change following imatinib treatment.

## 2. Materials and Methods

We performed low input RNA-seq on bronchial brushing samples obtained from patients enrolled in the KIT Inhibition by Imatinib in Patients with Severe Refractory Asthma (KIA) trial [1]. Ten imatinib-treated patient samples and 14 placebo-treated patient samples were analyzed. We defined clinical response to imatinib treatment as an increase of ≥5% between baseline and post-imatinib FEV_1_ measurements.

To identify changes in gene expression by imatinib treatment, we first performed differential expression (DE) analysis (paired *t*-test) on normalized data from all paired samples between pre-and post-imatinib and pre- and post- placebo treatments. To account for false positive expressions related to placebo effects, we removed genes that were significantly DE and had the same direction of effect in both placebo and imatinib-treated groups. The Benjamini-Hochberg method was used to control the false discovery rate.

We, then, constructed personalized perturbation profiles (PEEPs) from the gene expression data using genes uniquely expressed in imatinib-treated patients [9]. PEEP methodology addresses the genetic heterogeneity of complex diseases, such as asthma, by characterizing gene expression patterns at the individual patient level. For each individual in the imatinib treatment (case) group, PEEP compares the expression level of a gene to a reference distribution of the expression level of that same gene in the placebo (control) group. The resulting deviation, calculated as a z-score, is positive if the gene is overexpressed with respect to the control distribution, and negative otherwise. This analytic approach has been validated in asthma, among other chronic diseases [9]. Using PEEP, we identified post-imatinib gene expression changes at the level of single individuals, compared to baseline gene expression in the pre-imatinib patient population. Based on the PEEP analysis results, we then conducted a hierarchical clustering using the average method and Euclidean distance metric. We examined the association of these profiles with patient phenotypes with and without meaningful clinical improvements in FEV_1_.

Next, to reduce the false positives and account for fluctuations in expression levels, only genes with absolute z-score of 2 or above are selected as differentially expressed in the individual profiles. We identified biological processes upregulated or repressed across the imatinib-treated population using pathway enrichment analysis (Enrichr, g:Profiler) on genes with z-scores above 2 and z-scores below −2 [10]. We used a functional protein-protein interaction (PPI) network (HumanNet-FN) [11] to identify a disease module (gene set related to the imatinib response phenotype that forms a connected component) as a means of identifying potentially relevant pathways, rather than individual genes, that might not otherwise be detected with DE alone. Finally, we measured the distances between genes of these pathways to genes from patients with greatest increases in FEV_1_, and to genes from patients without improvement in FEV_1_. Genes in close proximity within protein-protein interaction networks have been found to demonstrate similar biological functions [12].

## 3. Results

### 3.1. Personalized Perturbation Profile (PEEP) Analysis

At baseline, there were no significant clinical differences between the imatinib and placebo-treated subsets with regards to demographics, FEV_1,_ markers of airway hyperresponsiveness, inflammation, and Asthma Control Questionnaire (ACQ-6) score (Table 1).

**Figure 1:**
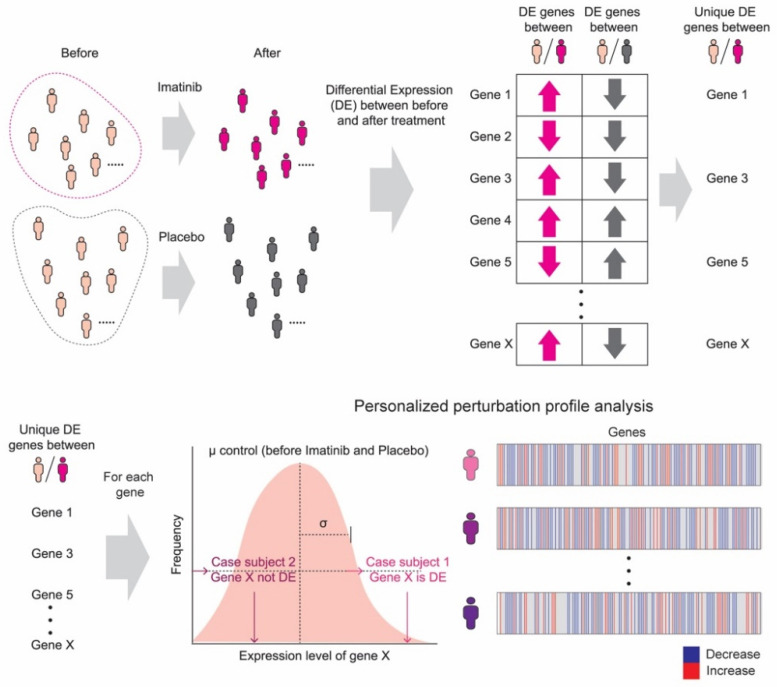
Personalized perturbation profile (PEEP) analysis pipeline. PEEP compares gene expression changes post-treatment at the level of single individuals, to baseline gene expression in a pre-treatment patient population.

**Figure 2:**
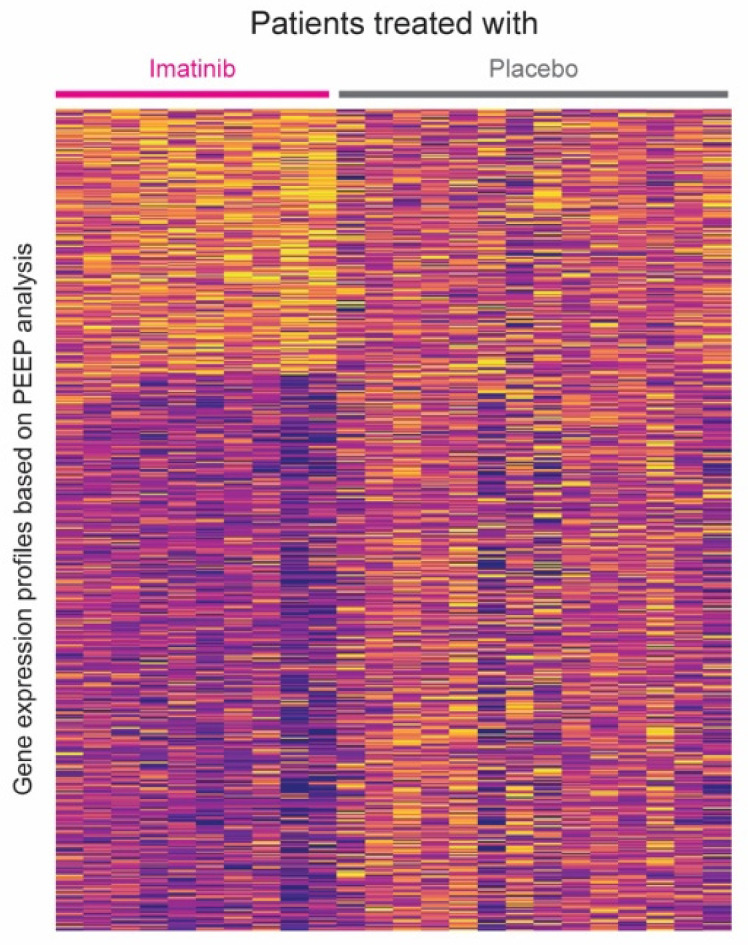
Gene expression profiles based on PEEP analysis. Gene expression profiles are compared based on imatinib or placebo-treated group.

Paired *t*-testing from the RNA-seq data yielded 557 genes that were differentially expressed in the imatinib treated group at a nominal level of significance (*p*-value ≤ 0.05) (Supplementary Table S1). PEEP analysis on these genes resulted in a unique expression profile for each patient (*n* = 10) (Figure 1). Differences in the gene expression profiles based on PEEP analysis between imatinib and placebo-treated patients is depicted in Figure 2.

### 3.2. Hierarchical Clustering Based on PEEP

**Figure 3:**
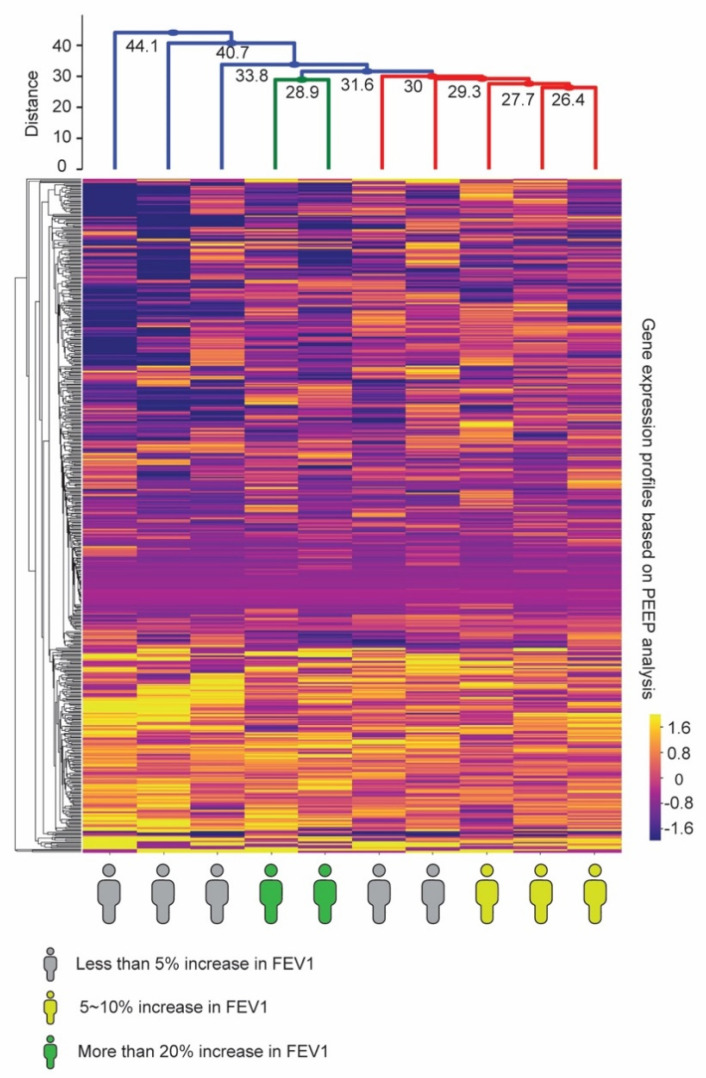
Individual patient gene expression profiles are associated with forced expiratory volume in 1 s (FEV_1_) improvement on imatinib and are further distinguished by the degree of FEV_1_ change. Patients in yellow demonstrated a weak (5–10%) increase in FEV_1_, while patients in green demonstrated a strong (>20%) improvement in FEV_1_.

Unsupervised clustering on the gene expression profiles of the 557 genes based on the PEEP analysis identified that patients with FEV_1_ improvement on imatinib (*n* = 5) shared similar profiles (Figure 3). Clustering yielded distinct groups of patients corresponding to percent change in FEV_1_: Two patients (green; entitled strong responders relative to the other groups) had an improvement greater than 20% while three patients (yellow; entitled weak responders) had a 5–10% increase in FEV_1_ and five patients (in gray) had <5% response (entitled non-responders). The FEV1 response is a continuous variable [13]; therefore cut-offs for analyses were objectively based on the clustering results and not pre-specified. In an exploratory analysis, we found that the average decrease in serum tryptase during between pre- and post-treatment visits was greatest among FEV_1_ strong responders, lower for weak responders, and least among non-responders (Supplementary Figure S1). 

Profiles of weak responders shared no common genes with z-scores above 2 or below −2 and there were no common up- or down-regulated pathways. Similarly, imatinib-treated patients without improvement in FEV_1_ shared no common up- or down-regulated biological processes, suggesting that the lack of drug response in these patients may stem from heterogenous factors.

In contrast, we found four genes were commonly repressed across the strong responders compared to the imatinib-treated population in the PEEP analysis: MPV17L2 (*p* = 0.02), CNIH2 (*p* = 0.02), AP1M2 (*p* = 0.04), PTPMT1 (*p* = 0.04). We found that these strong responders uniquely shared multiple downregulated mitochondrial-related pathways, such as mitochondrial ribosome assembly, gene expression, and localization (Supplementary Table S2). Among weak responders, and non-responders, none of these pathways were significantly affected.

### 3.3. Shortest Distance Analysis to Mitochondrial Genes

**Figure 4:**
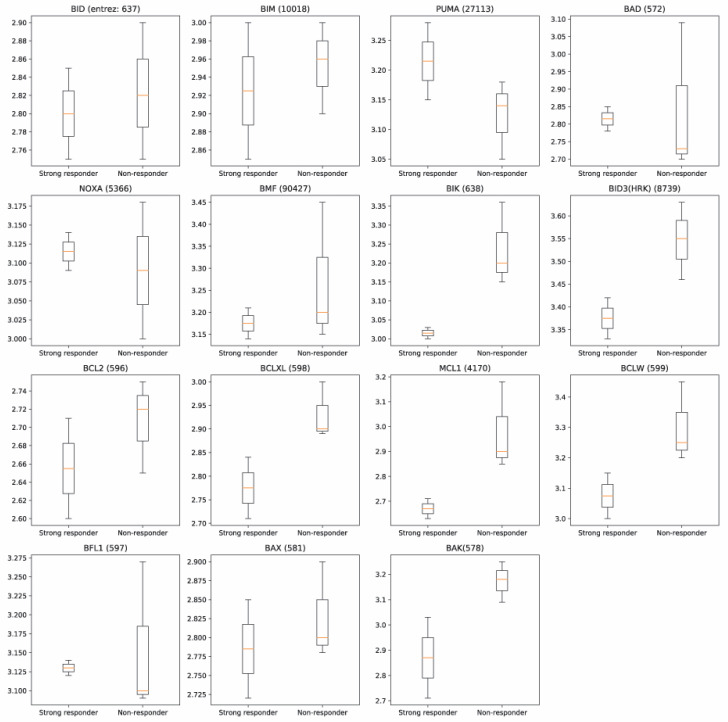
In the functional protein-protein interaction network, Bcl-2 family genes are closer to differentially expressed genes of imatinib-treated patients with strong improvement in forced expiratory volume in 1 s (FEV_1_) than to non-responders. Whisker plots represent the distance of the genes from each other. Genes in close proximity within protein-protein interaction networks have been found to demonstrate similar biological functions.

In the functional PPI mitochondrial genes in the Bcl-2 family measured in closer proximity to the shared, repressed genes of strong responders, than to genes of imatinib non-responders. In particular, mitochondrial genes BIK, BID3, BCLXL, MCL1 and BCLW of the anti-apoptotic subgroup measured closest to strong responders’ genes (Figure 4).

## 4. Discussion

In this pilot study, we applied systems biology approaches to clinical trial data to study the heterogeneous response of patients with severe asthma to imatinib treatment. Applying PEEP to the genomic data on a subset of trial participants generated individual-level gene expression profiles of imatinib response. An analysis of these profiles identified a set of pathways and related genes that distinguished strong responders from weak responders, and from non-responders, based on FEV_1_ change with imatinib. Patients with improved lung function after treatment showed a set of uniquely repressed mitochondrial genes in their bronchial epithelium brushings.

There has been increasing attention on mitochondrial dysfunction in airway disease [14]. Multiple features of mitochondrial function and structure have been implicated in the pathophysiology of asthma [15,16]. Altered homeostasis of mitochondrial biogenesis may lead to airway remodeling (e.g., increase in bronchial smooth muscle) that is characteristic of severe asthma [17]. Similarly, reduced mitochondrial membrane potential and metabolic activity may play a role in fibroblasts and other cell populations contributing to the pathogenesis of fibrosis in severe asthma [18,19]. The non-eosinophilic phenotype is more common in severe asthma, suggesting that mitochondrial mechanisms may be particularly relevant in this domain. Mitochondrial membrane permeability and stability responds to environmental stressors [14], as well as to mitochondrial products released from damaged mitochondria, which may be important in inflammasome-mediated mechanisms of non-eosinophilic asthma [20]. Increasingly, bioinformatics [19] and omics [21] approaches are being used to elucidate the impact of genetic differences in mitochondrial dysfunction and airway disease. Our findings add to this growing literature and its relevance to novel therapeutics [22]. Mitochondrial genes in the Bcl-2 family measured closer to the genes of strong responders in our PPI network. Bcl-2 proteins have previously been reported to be involved in mitochondrial stability [23], and mediate imatinib chemosensitivity [24]. The close proximity of these genes within the PPI network further supports the role of these pathways in airway response to imatinib.

The limitations to our pilot analysis include that we were only able to perform RNA-seq on a subset of responders and non-responders in the KIA trial. While our subjects were representative of trial participants, the relatively small sample size of many clinical trials like ours, and the heterogeneity of clinical response to treatment, constitute a challenge for traditional group-wise analyses. We accounted for the latter limitation by estimating individual-level variation with PEEP. Specifically, the PEEP study design uses two sets of controls in a robust analysis: Placebo-treated patients to eliminate false positive results and the individual-level comparison to eliminate between person variability. This approach likely contributes to our ability to see significant results despite a small sample size. Our use of PEEP highlights its potential for application in omics analysis of larger cohorts from future clinical trials to develop personalized phenotypes and targeted treatment—particularly in traditionally poorly understood disease states such as non-eosinophilic asthma.

## 5. Conclusions

Our results suggest a new role for mitochondrial pathways in airflow limitation in non-eosinophilic severe asthma and highlight these pathways as potential therapeutic targets. Large-scale clinical trials using imatinib are planned, setting the stage for the validation of these findings and addressing a pressing clinical need for additional treatment options for severe asthma [25].

## Figures and Tables

**Table 1:** Baseline demographic characteristics * of patients included in bronchial brushing analysis.

Characteristic	Imatinib (N = 10)	Placebo (N = 14)
Age (years)	44 ± 7	41.7 ± 12.3
Female sex (no. %)	60%	50%
White race ^ (no. %)	50%	35.7%
Log_2_ methacholine PC_20_^‡^	1.29 ± 1.5	1.47 ± 1.3
FEV_1_—Percent of predicted	71.5 ± 12.4	68.3 ± 11.7
FeNO ^‡^—ppb	23.4 ± 11.5	32.4 ± 11
Peripheral eosinophil count, per cubic millimeter	6.6 ± 7.2	2.95 ± 7.2
BAL neutrophils ^#^	4.2 ± 8.8	2.3 ± 5.6
BAL eosinophils ^&^	0.79 ± 0.81	0.54 ± 0.88
ACQ-6 score ^†^	1.8 ± 1.1	2.2 ± 1

* Plus–minus values are means ±SD. There were no significant differences between the groups using a two-tailed *t*-test with significance set at *p* < 0.05. PC20 provocative concentration of methacholine ± causing a 20% decrease in FEV1. FEV1 forced expiratory volume in 1 s. FeNO fraction of exhaled nitric oxide. BAL denotes bronchoalveolar lavage. ^^^ Race was reported by the patient. ^‡^ Data were available for 9 patients in the imatinib group. ^#^ Data were available for 8 in imatinib group and 12 in placebo group. ^&^ Data were available for 7 in imatinib group and 12 in placebo group. ^†^ Scores on the six-item Asthma Control Questionnaire (ACQ-6) range from 0 to 6, with lower values denoting better asthma control. The minimally important difference is 0.5.

## Supplementary Materials

The following are available online at https://www.mdpi.com/2075-4426/11/4/240/s1, Table S1: Genes differentially expressed in imatinib-treated patients, Table S2: Pathway enrichment analysis results for patients with >20% improvement in forced expiratory volume in 1 s (FEV_1_) after imatinib treatment, Figure S1: Change in serum tryptase by FEV1 response.
