# Supplementary material for: Systems Approaches to Treatment Response to Imatinib in Severe Asthma: A Pilot Study
Source: J Pers Med. 2021 Mar 25;11(4):240. doi: 10.3390/jpm11040240 (PMC8064376; doi:10.3390/jpm11040240)
Supplement: Supplementary file 1 [file jpm-11-00240-s001.zip › Suppl_Table2_enrichment_analysis_of_two_responders.docx]

**Supplementary Tables S2A-B.** Pathway enrichment analysis results for patients with >20% improvement in forced expiratory volume in 1 second (FEV_1_) after imatinib treatment

A.

| Term Names (Gene Ontolgy: Biological processes) | padj |
| --- | --- |
| Mitochondrial ribosome assembly | 1.592 × 10^-4^ |
| Mitochondrial gene expression | 3.048 × 10^-3^ |
| Regulation of protein targeting to mitochondrion | 3.851 × 10^-3^ |
| Mitochondrion localization | 3.989 × 10^-3^ |
| Mitochondrion electron transport, NADH to ubiquinone | 5.723 × 10^-3^ |
| Regulation of protein processing involved in protein targeting to mitochondrion | 6.621× 10^-3^ |
| Negative regulation of protein processing involved in protein targeting to mitochondrion | 6.621× 10^-3^ |
| Mitochondrion organization | 8.276 × 10^-3^ |
| Regulation of establishment of protein localization to mitochondrion | 8.440 × 10^-3^ |
| Regulation of mitochondrial membrane potential | 8.783 × 10^-3^ |

B.

| Term Names (Gene Ontolgy: Biological processes) | padj |
| --- | --- |
| Mitochondrial ribosome assembly | 2.035× 10^-4^ |
| Mitochondrion organization | 4.860 × 10^-3^ |
| Mitochondrial translation | 6.320 × 10^-3^ |
| Mitochondrial gene expression | 7.810 × 10^-3^ |
| Positive regulation of mitochondrial translation | 1.976 × 10^-2^ |
| Regulation of mitochondrial translation | 2.589× 10^-2^ |
| Regulation of mitochondrial gene expression | 2.691× 10^-2^ |
| Positive regulation of protein targeting to mitochondrion | 2.889 × 10^-2^ |
| Regulation of protein targeting to mitochondrion | 3.541 × 10^-2^ |
| positive regulation of establishment of protein localization to mitochondrion | 4.223 × 10^-2^ |
