# Supplementary figures and images for: Systems Approaches to Treatment Response to Imatinib in Severe Asthma: A Pilot Study
Source: J Pers Med. 2021 Mar 25;11(4):240. doi: 10.3390/jpm11040240 (PMC8064376; doi:10.3390/jpm11040240)

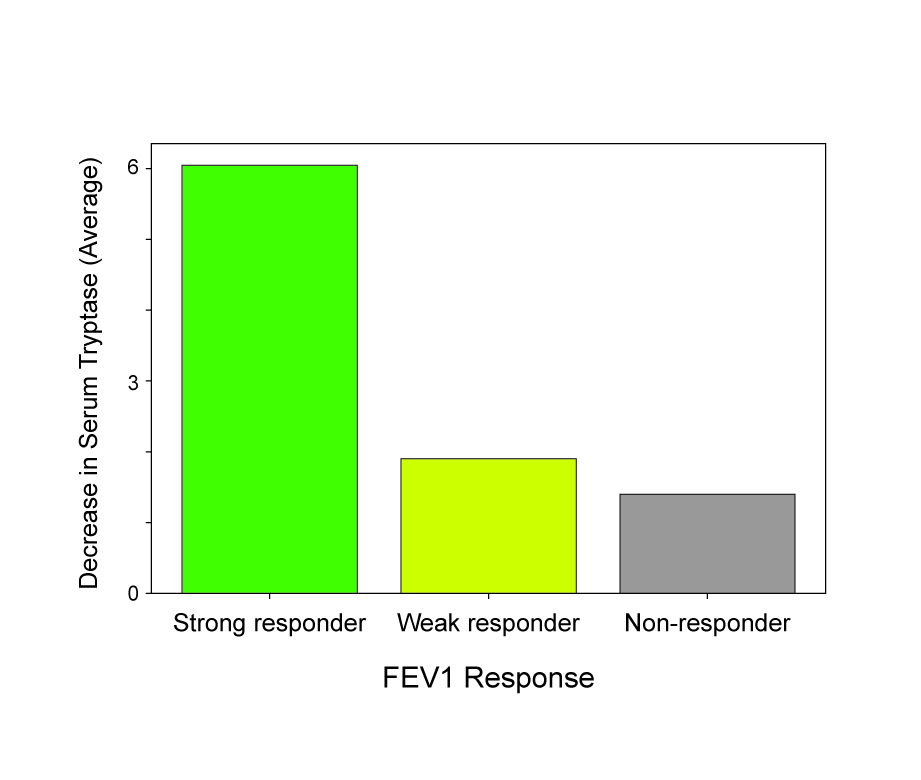

Supplement: Supplementary file 1 [file jpm-11-00240-s001.zip › Suppl_Figure_S1_tryptase.jpg]
